# A Study on Effects of Cognitive Function on Driving Behavior in Older Adult Drivers

**DOI:** 10.1002/alz70861_108509

**Published:** 2025-12-23

**Authors:** Mio Suzuki

**Affiliations:** ^1^ Tokai University, Hiratsuka, Kanagawa Japan

## Abstract

**Background:**

Accidents caused by elderly drivers continue to be a major issue, accounting for about 15% of all accidents in the Corona Disaster. Currently, cognitive function tests play a major role in determining whether elderly drivers can continue to drive. However, driving behavior, which consists of cognition, judgment, and operation, is considered to play an important role not only in cognitive function but also in physical ability.

Therefore, we attempted to obtain basic knowledge on the relationship between physical functions and driving among elderly drivers.

**Method:**

Subjects drove as instructed by a driving school instructor riding in the front passenger seat and experienced cranks, lane changes, right and left turns, and parking.

Driving evaluations were analyzed based on whether or not an event was observed during the driving experiment (0/1). Observation of events was recorded at the discretion of the driving school instructor.

After driving, cognitive function was tested, body composition was measured with a body composition analyzer, and physical measurements were taken. Cognitive function was investigated using a touch‐screen ADAS. The experimental period was from June 2022 to July 2023, and the valid sample size was 65 (average74.8, S.D.2.56 years old, 50 males and 15 females).

**Results:**

First, there were no significant differences in body composition by cognitive function.

On the other hand, subjects with MCI or suspected dementia had weaker grip strength in the right hand, and spent more time walking slowly and walking at the fastest speed.

This may indicate that the muscles of the entire body, including the right hand, which should be used frequently, are weak.

**Conclusions:**

It should be noted that the sample size for dementia or MCI is not large enough, but we found several relationships between cognitive and physical function and driving.

In particular, the fastest gait may influence the stopping behavior. The balance of physical abilities may influence driving maneuvers. Strength of exertion may influence left‐turn behavior. Dementia and MCI are not good for those functions. Both of these factors are very important for safe driving, so it is important to maintain physical and cognitive functions to improve quality of life.

